# Adaptive optimisation of a generalised phase contrast beam shaping system

**DOI:** 10.1016/j.optcom.2014.12.059

**Published:** 2015-05-01

**Authors:** F. Kenny, F.S. Choi, J. Glückstad, M.J. Booth

**Affiliations:** a Centre for Neural Circuits and Behaviour, University of Oxford, Mansfield Road, Oxford OX1 3SR, United Kingdom; b DTU Fotonik, Department of Photonics Engineering, Ørsted Plads 343, Technical University of Denmark, DK-2800 Kgs. Lyngby, Denmark; c Department of Engineering Science, University of Oxford, Oxford OX1 3PJ, United Kingdom

**Keywords:** Phase contrast, Adaptive optics, Spatial light modulators

## Abstract

The generalised phase contrast (GPC) method provides versatile and efficient light shaping for a range of applications. We have implemented a generalised phase contrast system that used two passes on a single spatial light modulator (SLM). Both the pupil phase distribution and the phase contrast filter were generated by the SLM. This provided extra flexibility and control over the parameters of the system including the phase step magnitude, shape, radius and position of the filter. A feedback method for the on-line optimisation of these properties was also developed. Using feedback from images of the generated light field, it was possible to dynamically adjust the phase filter parameters to provide optimum contrast.

## 1 Introduction

**Fig. 1:**
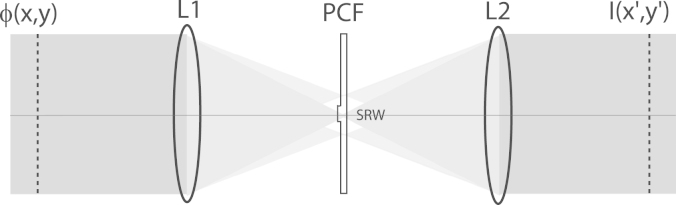
Phase contrast system based on a 4f system. A phase distribution, ϕ(x,y), is Fourier transformed using a convex lens and a phase contrast filter (PCF) shifts the zero-order diffracted spot with respect to the rest of the diffraction pattern. After re-collimation this phase shifted spot forms a synthetic reference wave (SRW). $I(x^{\prime },y^{\prime })$ represents the interference between the SRW and the higher spatial frequency components.

Phase distributions can be converted into intensity distributions by means of a common path interferometer. This concept was used in Zernike's phase contrast microscope [1], which can be used to observe phase fluctuations introduced by transparent or semi-transparent specimens. A phase contrast system usually constitutes a 4f system where the zero order or DC component of the Fourier transform of the phase distribution is phase-shifted by π/2 in order to generate a synthetic reference wave (SRW). Once the beam is re-collimated, the SRW interferes with the spatially varying phase fluctuations, and an interferogram can be observed at the output plane. This is illustrated in Fig. 1, where ϕ(x,y) represents a phase disturbance across the beam, and $I(x^{\prime },y^{\prime })$ represents the observation plane.

While Zernike phase contrast is useful for visualising small phase fluctuations, the case of generalised phase contrast (GPC) allows for the conversion of larger phase fluctuations to intensity distributions [2]. It is advantageous to use GPC for the generation of smooth continuous intensity distributions [3]. Even when the phase modulation element is pixelated, such as when using a liquid crystal spatial light modulator, the optical transfer function of the 4f system will provide a blurring effect, which results in a smooth intensity distribution. In addition to this, GPC provides efficient conversion of the input beam to a desired intensity distribution; efficiencies of up to 85% have been reported for binary GPC of a Gaussian beam [4], while efficiencies of 74% have been reported for the generation of greyscale intensity patterns [5]. With these properties GPC has uses in applications such as targeted photo-activation of neurons [6,7], optical trapping [8,9], and quantitative measurement of an unknown phase distribution [10]. In addition to being used for intensity shaping of monochromatic beams, GPC has also been extended to broadband beams [11].

Another common method used to generate custom intensity distributions is holographic beam shaping. This technique uses phase distributions in the entrance pupil of a lens to generate an intensity pattern at the focus of the lens [12]. The appropriate pupil phase pattern can be found using methods such as the Gerchberg–Saxton algorithm [13]. While this is a powerful technique, speckle can become a problem in the focal region of the lens. GPC can overcome this difficulty as it is based around a 4f imaging system, where the output intensity distribution is conjugate to the phase modulation applied at the entrance pupil [14].

A number of other techniques have been developed for intensity shaping of both coherent and incoherent sources. These include those that use diffractive optical elements to generate intensity distributions to generate flat-top illumination [23,25,24] or more complex illumination patterns [22]. Arrays of microlenses can also be used for intensity shaping [19,20], as is described in [26], where Köhler illumination was generated using two microlens arrays. A technique using two aspheric lenses to convert a Gaussian beam to a flat-top beam has also been described [21].

A spatial light modulator is often used to generate the initial phase distribution in the entrance pupil of the system, while the phase contrast filter (PCF) can be constructed from a machined plate of glass, where a small area in the centre is machined such that it is thinner than the surrounding glass. Alternatively, the optical path length through the central region of the filter can be increased by the addition of a layer of material. These filters are usually static, and their parameters are chosen in advance based on theoretical calculations [2,9,6].

In this work, we used two passes on a single SLM. The first pass was used to control the pupil phase distribution, while the second generated the phase contrast filter. This provided further flexibility and stability to the system; in order to change any filter parameters no mechanical changes to the system were required, only adjustments of the phase pattern on the SLM. This setup also allowed control of the alignment of the GPC system by the optimisation of a contrast metric calculated from the output intensity distribution. This metric provided a measure of the quality of the operation of GPC setup and the suitability of the PCF parameters.

## 2 Background

Experimental implementation of GPC is based on a spatial filtering system [15], similar to that shown in Fig. 1, where the input phase distribution is controlled by a phase modulation device such as a spatial light modulator (SLM). Such device generates the input field, a(x,y), which, for a circular aperture, is represented as follows:

(1) $$a(x,y)=\mathrm{circ}(\frac{r}{\Delta r})\exp(i\phi (x,y)),$$

where (*x*,*y*) is the coordinate system defining positions across the beam at the entrance pupil of the system, $r=\sqrt{x^{2}+y^{2}}$, Δr is the radius of the pupil, and *ϕ* is the phase distribution across the beam. In the case of Zernike phase contrast, the phase distribution is approximated using the first terms of a Taylor expansion, and is valid for small phase disturbances. In the case of GPC, higher order terms in the expansion are taken into consideration, which means that GPC can be used to visualise larger magnitudes of phase than Zernike phase contrast [15].

After the phase distribution has been Fourier transformed by a lens, a phase contrast filter can be introduced, which phase shifts some of the zero-order diffracted light. This filter can be described using the following equation:

(2) $$H(f_{x},f_{y})=A[1+({\mathrm{BA}}^{-1}\exp(i\theta )-1)\mathrm{circ}(f_{r}/\Delta f_{r})],$$

where *f*_*x*_ and *f*_*y*_ are the spatial frequency coordinates in the Fourier domain and *f*_*r*_ is the spatial frequency radius ($f_{r}=\sqrt{\left(f_{x}^{2}+f_{y}^{2}\right)}$). *B* is the transmittance within the diameter of the phase contrast filter, while *A* is the transmittance for off-axis light. *θ* is the magnitude of the phase step of the PCF and $\Delta f_{r}$ is the spatial frequency radius of the filter. The central region of the PCF phase shifts a portion of the zero order of the Fourier transform with respect to the higher spatial frequency components that are diffracted off-axis.

Once the beam is re-collimated by a second lens, the phase shifted portion acts as a synthetic reference wave (SRW) that can interfere with the re-collimated higher order diffracted light. In this way the system acts as a common path interferometer which is similar to the Zernike phase contrast system. The output of the GPC system consists of the interference between the SRW and the higher spatial frequency components of the incident beam and is represented by the following expression [3]:

(3) $$I(x^{\prime },y^{\prime })=A^{2}{\left|\exp[i\phi (x^{\prime },y^{\prime })]\mathrm{circ}(\frac{r^{\prime }}{\Delta r^{\prime }})+|\overset{¯}{\alpha }|[{\mathrm{BA}}^{-1}\exp(i\theta )-1]g(r^{\prime })\right|}^{2},$$

where *I* is the intensity of the distribution at the output plane, $\left(x^{\prime },y^{\prime }\right)$ are the spatial coordinates ($r^{\prime }=\sqrt{\left(x^{\prime 2}+y^{\prime 2}\right)}$ ), $\overset{¯}{\alpha }$ is the average phase across the input phase distribution, and Δr is the radius of the aperture stop of the system. $g(r^{\prime })$ represents the synthetic reference wave; for a circular aperture and circular phase contrast filter it is defined as [3]

(4) $$g(r^{\prime })=2\pi \Delta r\int_{0}^{\Delta f_{r}}J_{1}(2\pi \Delta {\mathrm{rf}}_{r})J_{0}(2\pi r^{\prime }f_{r})\;{\mathrm{df}}_{r},$$

where $\Delta f_{r}$ is the spatial frequency radius of the phase contrast filter and all other quantities are as previously defined.

Eq. (3) amounts to a coherent sum between its first and second terms within the modulus brackets. A number of quantities govern the expected contrast in the intensity distribution, such as the phase step of the filter, *θ*, the spatial frequency radius of the filter, $\Delta f_{r}$, the average phase across the initial phase distribution, $\overset{¯}{\alpha }$, and the transmission coefficients of the filter, *A* and *B*.

In previous work, the filter has been machined from a piece of glass and was fixed after manufacture. As a result of this, the operation of the GPC system was constrained. The experimental system, which will be described in the next section, could control all aspects of the filter, and was therefore a suitable test-bed for experiments on the effects of varying its parameters. Control over the transmission of the filter could be attained by applying a high spatial frequency pattern to the SLM, such that reflected light would be diffracted outside the aperture of the collecting lens.

## 3 Experimental system

**Fig. 2:**
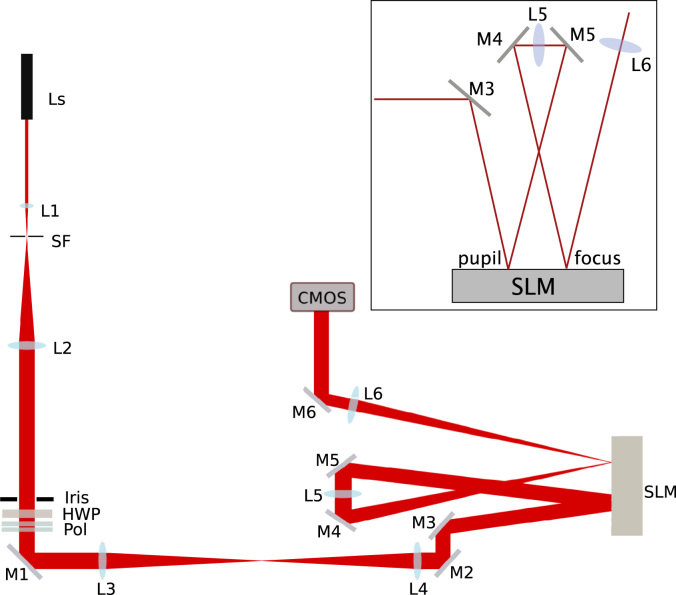
Schematic of the GPC system using two passes on a single SLM. The first SLM pass introduced a phase distribution across the beam while the second applied a phase shift to the centre of the Fourier transform of the input phase distribution. Ls is a diode laser, L1–L6 are lenses, M1–M6 are plane mirrors, SF is a spatial filter, HWP is a half wave plate, Pol is a polariser, SLM is a liquid crystal spatial light modulator (P512-0785 manufactured by Boulder Nonlinear Systems), and a CMOS camera was used as detector. The inset figure shows the design of the two-passes on the SLM in more detail.

The experimental system is depicted in Fig. 2. A diode laser (Edmund Optics #85-230 4.5 mW CW, *λ*=780 nm) was first spatially filtered and expanded using lenses L1 and L2 and the spatial filter (SF). An iris defined the size of the beam incident on the first pass of the SLM and was set to approximately 4 mm in diameter. This was imaged onto the SLM using lenses L3 and L4. The SLM was manufactured by Boulder Nonlinear Systems (model no. P512-785), and was capable of a full wave of modulation at 785 nm. It consisted of a 512×512 array of pixels of size 15 μm. Turning mirrors were used to direct the beam reflected off the SLM through lens L5, which focused the beam onto an adjacent portion of the SLM. The focal length of this lens was set at 500 mm in order to keep the angles of incidence and reflection onto the SLM less than 5°. The output intensity distribution was viewed on an 8-bit CMOS camera (Thorlabs DCC1545M). In this configuration, both a pupil plane and a focal plane could be simultaneously controlled using adjacent parts of the SLM.

**Fig. 3:**
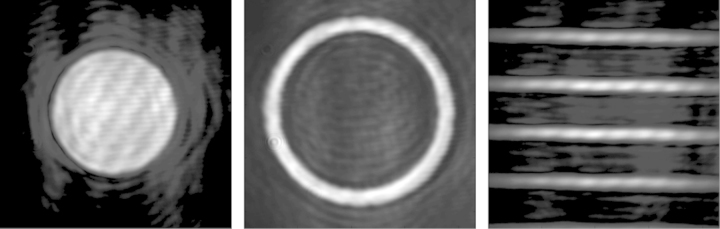
Example output images from the GPC system. Intensity distributions of a circle, annular aperture and a set of lines with 25% duty cycle were generated. The weak diagonal fringes visible across the images were due to the interference of a back reflection off a polariser in the optical system.

LABView software was written to control all aspects of the system controlled by the SLM, such as the input phase distribution and the filter parameters; the position of the filter could also be changed using this software. Three example output images from the system are shown in Fig. 3. These were obtained using input binary phase patterns. The intensity patterns produced were a circle, an annulus and a pattern of lines of spatial duty cycle 25%. These were binary patterns that were phase shifted by *π* with respect to the background. The images in Fig. 3 showed that the dual-pass SLM-based system was operating as a conventional GPC system.

As the system provided flexibility in the control of both the input phase distribution and the phase contrast filter, this permitted the implementation of a feedback scheme to optimise the parameters of the PCF. An image-based adaptive optics approach was used, whereby a quality metric derived from the contrast of an image of the output intensity was maximised. This approach has been used extensively for the correction of aberrations in microscopes and other optical systems [16].

## 4 Filter optimisation

Using the SLM to generate the phase contrast filter provided flexibility and programmability in controlling the filter's parameters. Parameters such as the magnitude of the phase step, diameter, shape and position of the filter could be controlled. This provided an advantage in the adaptive alignment and optimisation of the filter parameters for different experimental requirements.

We illustrate this capacity using a metric, which was chosen to assess the quality of binary-intensity GPC output patterns obtained from the system. First, a mask was generated from the desired intensity pattern, dividing the pixels into those inside and outside the mask, corresponding the bright and dark areas in the desired intensity patterns. The metric, *μ*_*contrast*_, was defined as the difference between the sum of the pixel values inside and outside the mask, denoted by *η*_*inside*_ and *η*_*inside*_ respectively; the bright areas were inside the mask, while the dark areas were outside

(5) $$\mu_{\mathrm{contrast}}=\sum \eta_{\mathrm{inside}}-\sum \eta_{\mathrm{outside}}$$

### 4.1 Filter diameter and phase step optimisation

The first two filter parameters to be optimised were the magnitude of the phase step and the radius of the filter. As each of the filter parameters were independent of each other, they could be optimised in turn. Two test phase distributions were used in the entrance pupil of the system, and applied using the first pass of the SLM. These were the circle and the set of lines shown in Fig. 3.

In optimising the radius and phase step of the filter, the system was first coarsely aligned by adjusting the position and radius of the filter with a phase step of *π*, which was an estimate of the optimal phase step. A routine was written in LabVIEW to vary the radius or phase step in turn, and then to record an image. The contrast metric was calculated for each value of the radius or phase step.

### 4.2 Filter diameter

**Fig. 4:**
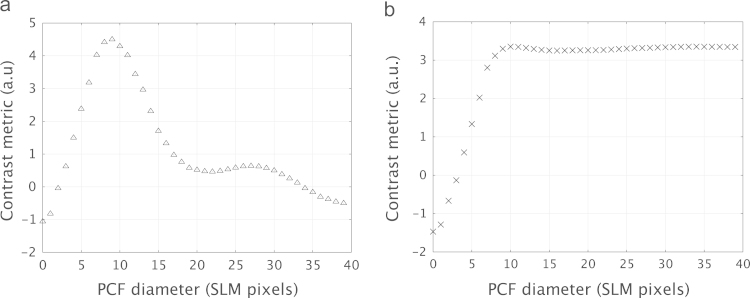
The calculation of the contrast metric was used to optimise the diameter of the phase contrast filter, which was controlled using second pass on the SLM. Metric curves were obtained for two intensity patterns, a circle (a), and a set of lines of duty cycle 25% (b).

**Fig. 5:**
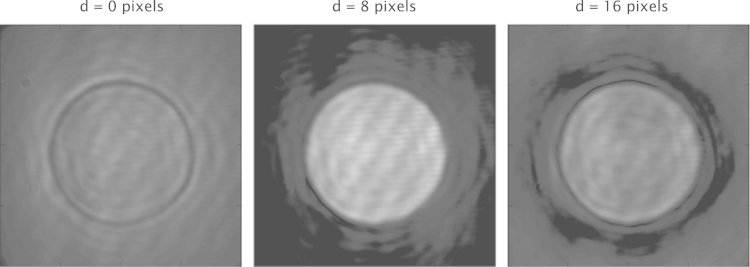
GPC images of a circle shown for different diameters of phase contrast filters, increasing from left to right. The PCF for the left image has no PCF present; there is little difference in intensity between pixels inside and outside the circle. The PCF for the central image has a more optimal diameter of 8 SLM pixels (pixel size=15 μm), while the PCF for the right image is larger than optimal at 16 pixels.

Using the circular phase pattern, the optimal diameter for the filter was found to be 9 pixels, which was equivalent to 135 μm as the pixel size of the SLM was 15 μm. This metric curve, depicted in Fig. 4(a) showed a clear peak indicating the optimal filter diameter for this pattern. Plotting a similar curve for the second input phase distribution gave a different value for the optimal filter diameter. The optimal filter diameter for the line pattern was 10 pixels, equivalent to 150 μm. The metric curve for this measurement is shown in Fig. 4(b). Example images from this experiment are also shown in Fig. 5. This figure shows the GPC image obtained using the circle pattern for different diameters of phase contrast filter. These qualitatively show a variation in the contrast of the output when the diameter of the PCF is changed.

Additionally, the contrast observed in the line pattern did not depend greatly on the filter diameter as long as the diameter was greater than ten pixels. This is different to the trend observed using the circle pattern, and can be explained if we consider the line pattern and its corresponding Fourier transform. The Fourier plane in this case will consist of the zero order in the region of the geometric focus, as well as a line of discretely space points representing the regular spatial frequency content of the pattern. The orientation of these points depends on the orientation of the line pattern in the input phase distribution. Little light will be present between these discrete points and, therefore, modifying the filter diameter up to the diameter corresponding to the first-order diffracted spots should have little effect on the measured contrast. The dependence of the optimum PCF diameter upon the desired output intensity illustrates the benefit of the adaptable GPC configuration.

### 4.3 Phase step

**Fig. 6:**
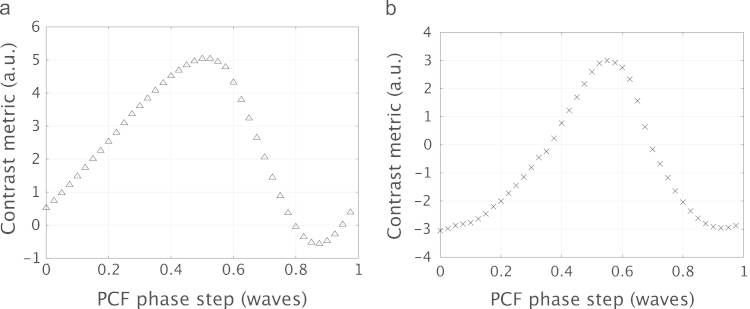
The calculation of the contrast metric was used to optimise the magnitude of the phase step, *θ* in Eq. (2), of the phase contrast filter, which was controlled using the second pass on the SLM. Metric curves were obtained for two intensity patterns, a circle (a), and a set of lines of duty cycle 25% (b).

The results for the optimisation of the phase step and the radius are shown in Fig. 6. The contrast metric was calculated for a set of input values of *θ*, varying in steps of 0.05 over one full wavelength. The SLM had previously been calibrated and linearised between crossed polarisers, such that the value of *θ* was known from the voltage applied.

The two desired output patterns were again used to illustrate the optimisation process, the circle and line patterns shown previously in Fig. 3. As shown in Fig. 6(a), the optimum phase step for the circular intensity distribution was found to be 0.525 waves, while for the line pattern, the optimal value of *θ* was 0.590 waves. The difference between these two values of phase step can be attributed to the $\overset{¯}{\alpha }$ term in Eq. (3). This term is related to the average value of the input phase distribution and directly affected the contrast in the interference described in Eq. (3). As the circle and line patterns have different average phases, it follows that their respective optimal values of *θ* would also be different. This has previously been discussed in [17], where the magnitude of the phase step could be matched to $\overset{¯}{\alpha }$ using a graphical phase chart and ternary phase distribution. $\overset{¯}{\alpha }$ can also be modulated in the pupil plane by including high spatial frequency patterns in the input image [18]. This diffracts some of the light off-axis so that it is not collected by the constituent lenses of the GPC system, and does not contribute to the final image.

### 4.4 Position optimisation

**Fig. 7:**
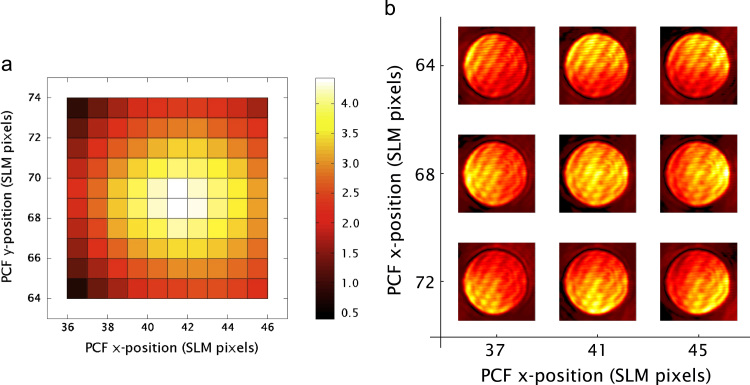
The calculation of the contrast metric was used to optimise the position of the phase contrast filter, which was implemented via a second pass on the SLM. The metric was measured over a 10×10 grid of positions on the SLM. This 2D plot (a) represents the contrast metric calculated for each of these positions, with the maximum at (41,68) indicating the optimal position of the PCF on the SLM. Example images (b) from the GPC system showing the effect of mis-alignments of the phase contrast filter. The central image was recorded when the filter was at its optimal position. When the filter was moved away from this position, gradients related to the displacement of the filter were visible in the recorded images.

The position of the filter was optimised by scanning the filter across a range of pixel positions and measuring the resulting contrast metric. First, the filter was coarsely positioned by eye; its position was then scanned over an 10×10 grid of SLM pixels centred on this coarse position. An image was recorded at each position and the contrast metric described in Eq. (5) was calculated. The resulting calculations of the metric are shown in Fig. 7.

The optimal position was found to be (68,41) as shown in the two-dimensional plot in Fig. 7(a). This was different to the initial manual position, approximately one pixel away in the vertical direction on the SLM. This corresponded to an difference in positioning of the filter of 15 μm, which is the size of one pixel on the SLM.

Fig. 7(b) shows the effect of mis-positioning of the phase contrast filter on the intensity distribution. In this figure, it can be seen that when the PCF was displaced from its optimal position, a gradient in the intensity distribution was observed. The orientation of this gradient was related to the direction of displacement of the filter. This property could also be used to develop a metric for the optimisation of the GPC system for both initial alignment and monitoring of the alignment of the system throughout an experiment. This effect is related to Eq. (3), which expressed the coherent sum of the SRW and the phase distribution. When the filter is positioned non-optimally, a misalignment between the SRW and the phase distribution occurs, which results in the observed gradient.

## 5 Conclusion

In this paper we have presented a dynamic generalised phase contrast system using two passes on a single SLM. The first pass was used to control the phase distribution across the beam, while the second pass coincided with the Fourier plane of this phase distribution and provided flexible control over the phase contrast filter. The controllable parameters of the filter included diameter, phase step size, and position. Although not shown here, the configuration also permitted modification of further PCF parameters including shape and coefficient of transmission. The latter could be controlled by applying a phase pattern of high spatial frequency in the filter plane.

Previous GPC systems had used fixed phase contrast filters, which placed constraints on the inputs and outputs of the system. The dynamic GPC system provided considerably more flexibility than a corresponding fixed phase contrast filter, as the parameters listed above could be controlled through software without physically adjusting any component of the system. With this, it was possible to develop a routine enabling adaptive alignment and optimisation of the phase contrast filter. An image contrast metric was introduced that measured the efficacy of the GPC system for any binary output image and provided a feedback criterion for the optimisation of the system configuration.

The contrast metric could be used to optimise the position of the phase contrast filter, thus removing any errors due to imprecise manual positioning. Results also suggested that the displacement of the filter from its optimal position had a predictable influence on the intensity distribution from the system. A gradient was observed in the images which depended on the distance and direction of the displacement of the filter. We note that the results presented in this paper were obtained using binary phase distributions. In the case of greyscale GPC, an adjustment would be made to the metric used, where a weighting would be applied to discrete areas of the GPC output. The weighting would depend on the expected GPC output.

Optimisation of the diameter of the filter gave different results depending on the input field. The metric curve for the circle had a clear maximum, while for the lines, once a threshold filter diameter had been reached there was little dependence on the size of the filter. In optimising the phase step of the filter, different optimal values were again obtained for the circle and the lines distributions. Dynamic control over the optimisation of the PCF provided the capacity to easily switch between these intensity distributions. This could be achieved using software to change the pattern applied to the SLM and is an advantage over GPC systems that use a static filter.
